# Beneficial effect of simvastatin on human umbilical vein endothelial cells gap junctions induced by TNF-α

**DOI:** 10.1080/19768354.2021.2023037

**Published:** 2022-01-16

**Authors:** Xiwen Ling, Siyuan Peng, Yaqin Xu, Fujiang Chu

**Affiliations:** a School of Biosciences and Biopharmaceutics, Guangdong Pharmaceutical University, Guangzhou, People’s Republic of China; b Guangdong Provincial Key Laboratory of Pharmaceutical Bioactive Substances, Guangdong Pharmaceutical University, Guangzhou, People’s Republic of China

**Keywords:** Connexin proteins, gap junctions, HUVECs, mechanism, simvastatin

## Abstract

Although simvastatin has been shown to inhibit vascular permeability, which might be amplified via gap junction intercellular communication (GJIC), the underlying mechanism of action remains unclear. In the present study, we investigated the effects and mechanisms of simvastatin on endothelial cells GJIC. Specifically, human umbilical vein endothelial cells (HUVECs) were stimulated with TNF-α (10 ng/mL) alone or in combination with simvastatin (5 µM), and their effects on vascular endothelial cell GJIC tested via the scrape loading/dye transfer (SL/DT) assay. Next, we performed immunofluorescence, real-time PCR and western blot assays to analyze expression of Cx37, Cx40 and Cx43 in HUVECs. Results showed that GJIC activity in HUVECs was markedly elevated in HUVECs treated with TNF-α in combination with simvastatin. In addition, simvastatin treatment significantly upregulated expression of Cx37 and Cx40 but downregulated Cx43 mRNAs and proteins. Taken together, these marked changes indicated that simvastatin exerts its regulatory effects on gap junction function by upregulating Cx37 and Cx40 and downregulating Cx43 expression.

## Introduction

Previous studies have shown that endothelial permeability increases and many inflammatory mediators contribute to elevated vascular permeability during early stages of atherosclerosis development (Lee 2005; Sitia et al. 2010). Moreover, endothelial permeability is reportedly affected by gap junction intercellular communication (GJIC) between neighboring endothelial cells (Figueroa et al. 2004; He et al. 2018). Gap junctions are protein channels in the cell membrane made of connexin molecules (Yeh et al. 2006). Connexin37 (Cx37), Cx40, and Cx43 are known to be expressed in endothelial cells at various sites along the vascular tree in mammals (Yeh et al. 2000; Yeh et al. 2003). Particularly, Cx37 and Cx40 have atheroprotective properties, while Cx43 appears to be pro-atherogenic (Meens et al. 2013; Peng et al. 2015). Simvastatin is a cholesterol-lowering drug that inhibits the HMG–Coenzyme A reductase enzyme. Findings from previous studies, conducted under low and normal cholesterol conditions, have shown that statins have both anti-inflammatory and lipid-lowering effects (McGown and Brookes 2007). Although recent investigations have demonstrated that simvastatin can improve endothelial function (O’Driscoll et al. 1997) and inhibit vascular permeability (Miyahara et al. 2004), its inhibitory effects on common inflammatory mediators via reduction of endothelial GJIC remains unclear. Tumor necrosis factor-alpha (TNF-α) plays an important role in orchestrating inflammatory responses in the vascular endothelium (van Rijen et al. 1998). Notably, human umbilical vein endothelial cells (HUVECs), a primary endothelial cell line, have been extensively used to study regulation of human endothelial cells genes, such as Cx37, Cx40 and Cx43 (Johnson and Nerem 2007).

The purpose of the present study was to investigate the effects and underlying mechanisms of simvastatin action on endothelial cells GJIC using an *in vitro* endothelial cell model. Specifically, HUVECs were stimulated using TNF-α (10 ng/mL) alone or in combination with 5 µM simvastatin, and their effects on vascular endothelial cell GJIC tested via the scrape loading/dye transfer (SL/DT) assay. In addition, patterns of Cx37, Cx40 and Cx43 expression in HUVECs were analysed via immunofluorescence, real-time PCR and western blot analyses.

## Materials and methods

### Cell line and treatment

**Figure 1.**
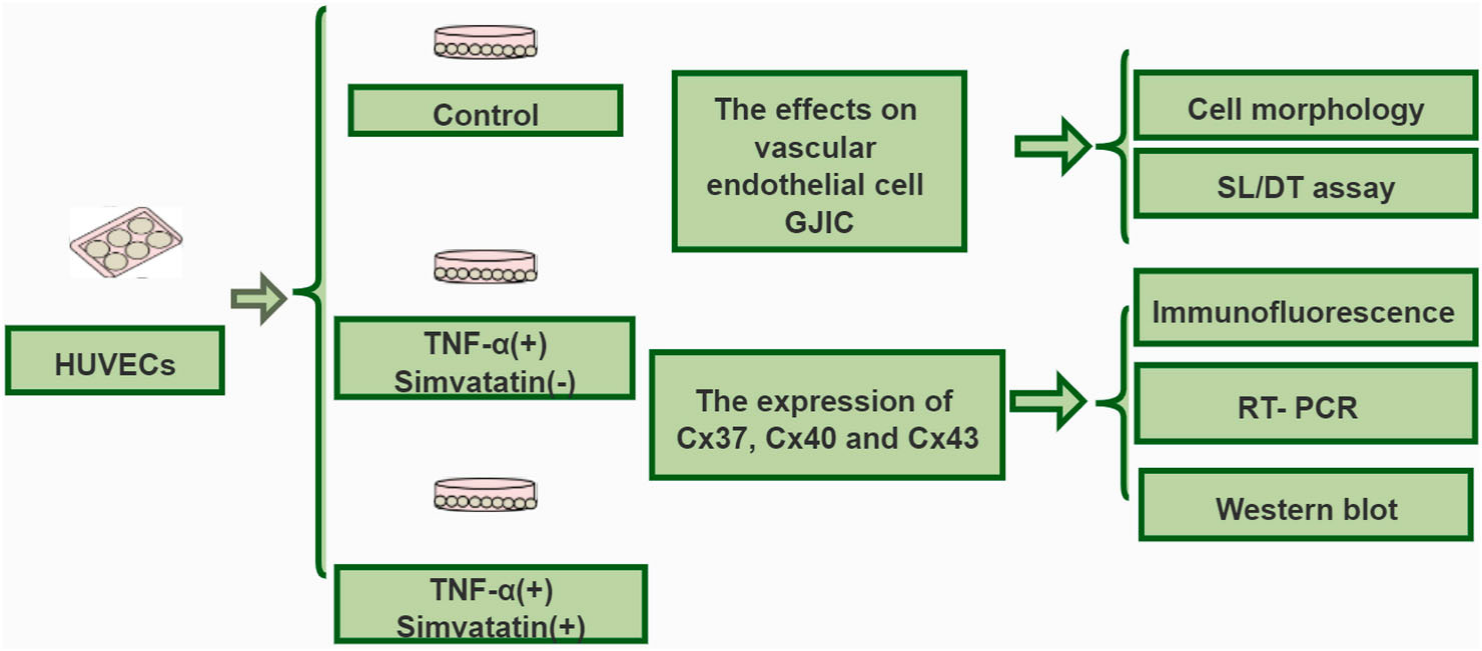
Schematic representation of the experimental design adopted in this study.

HUVECs were provided by Prof. H. L. Sun (School of Clinical Medicine of Guangdong Pharmaceutical University, China). Eighth-passage HUVECs were seeded in 6-well plates, at a density of 4 × 10^5^ cells/well and grown to 80% confluence. Subsequently, the cells were cultured in fresh medium (serum-free media) supplemented with TNF-α (10 ng/mL) alone or in combination with 5 μM simvastatin and incubated for 24 h at 37°C (Zapolskadownar et al. 2004). Stock solutions for simvastatin (Sigma, S6196) and TNF-α (Peprotech, 300-01A), 0.5 and 0.57 nM, respectively, were prepared. Prior to the experiment, these stock solutions were mixed with 0.5% DMSO, to obtain final working concentrations of 5 μM simvastatin and 10 ng/mL TNF-α. In addition, control cells across all experiments were treated with 0.5% DMSO, which did not affect cellular responses to simvastatin and/or TNF-α. GJIC in HUVECs was assessed by scrape loading/dye transfer (SL/DT). Next, we performed immunofluorescence, real-time PCR and western blot assays to determine expression patterns for Cx37, Cx40 and Cx43. The experimental process followed in this study is illustrated in Figure 1.

### Scrape loading/dye transfer (SL/DT) assay

Cell morphology was observed under a white light microscope (200× magnification). The SL/DT assay was used to measure gap junctional intercellular communication (GJIC) activity, and investigate the transfer of fluorescent Lucifer yellow dye from one cell into adjacent ones through functional gap junctions as previously described (Sundaram et al. 2011; Lu et al. 2018). Briefly, confluent density-inhibited cells were cultured in 60 mm polystyrene dishes, rinsed with CaMg-PBS, then mixed with 0.5% rhodamine-dextran and 0.5% Lucifer yellow in CaMg-PBS. The cell monolayer was scraped off, using a scalpel blade, and kept in the dark for approximately 3 min. The dye solution was then decanted, and the monolayer rinsed three times with CaMg-PBS. Subsequently, the cells were fixed with 4% (v/v) formalin, then subjected to fluorescence microscopy to observe the spread of the dye from wounded to adjacent intact cells. Distances between the transfer front and the scrape line, of each group on both sides of the scrape, were measured and calculated (Ke et al. 2013). Sizes of the fluorescence area along the scrape line on monolayers were quantified using Image pro plus software (Media Cybernetics, Georgia. USA) to reveal levels of GJIC.

### Immunofluorescence detection

Immunofluorescence assay was performed as described by Le Gal et al. (2014). Briefly, confluent cells grown on coverslips were fixed with 4% paraformaldehyde for 20 min, washed thoroughly with phosphate buffer saline (PBS), then blocked with 10% FBS and 0.3% Triton X-100 in PBS for 1 h. Next, the cells were incubated overnight at 4°C with the following antibodies: anti-Cx37 antibody (rabbit polyclonal Cx37 antibody raised against human; Cat. No. SAB4501180, Sigma-Aldrich), anti-Cx40 antibody (rabbit polyclonal Cx40 antibody raised against human; Cat. No. SAB1304973, Sigma-Aldrich) and anti-Cx43 antibody (rabbit polyclonal Cx43 antibody raised against human; Cat. No. C6219, Sigma-Aldrich). Then cells were then counter stained with secondary antibody conjugated with FITC (goat polyclonal antibody raised against rabbit; Cat. No. F0382, Sigma-Aldrich) for 1 h, and the nucleus stained with 1 µM diisopropylamine (DIPA) (Cat. No. 386464, Sigma-Aldrich). Finally, the cells were mounted and observed under a Leica fluorescence microscope with a ×20 objective. Fluorescent intensity for Cx 37, Cx 40 and Cx 43 in the cells were analyzed using Image J software, with nuclei staining by DAPI used to normalize the fluorescence intensity.

### Real-time PCR

Gene expression analysis was performed via quantitative real-time polymerase chain reaction (qRT-PCR) as described by Cervellati et al. (2011). Briefly, total RNA was extracted using the Trizol reagent, according to the manufacturer’s instructions, its concentration determined using a nanodrop spectrophotometer, and quality checked via agarose gel electrophoresis. qRT-PCR was performed using the iQTM SYBR Green Supermix (Bio-Rad), on a MyiQ Single-Color Real-Time PCR Detection System (Bio-Rad), targeting specific genes whose oligonucleotide primers are outlined in Table 1. Relative gene expression was normalized to that of *β*-actin, using the 2-ΔΔCT method against the control.

**Table 1.** List of oligonucleotide primers used for real-time PCR analysis.

Gene(GeneBank ID)	Primer sequence (5′→3′)	Tm (℃)	Product size (bp)
Cx37 NM_021654	Antisense: GAAGAAGTGGTCGTAGCA, Sense: AGGAGTAGAAGGGAAAGC	53.5	314
Cx40 NM_019280	Antisense: CAATCTTCCCGTTCACCT-3′, Sense: TCTCCCACATTCGTTACTG	53.1	213
Cx43 NM_012567	Antisense: AGAGCACTGACAGCCACA, Sense: TCCAAGGAGTTCCACCAA	52.9	156
β-actin NM_031144	Antisense: TCCTTCTGACCCATACC, Sense: TTTGTGCCTTGATAGTTCG	55.6	260

### Western blot assay

Detection of protein expression was performed as described by Cervellati et al. (2011). Briefly, total proteins were extracted by lysing cells in the RIPA buffer containing 150 mM NaCl, 5 mM EDTA, 1% NP40, 2 mM PMSF and 50 mM Tris-HCl, pH 7.4 or SB buffer containing 20% SDS, 0.1 M Tris-HCl, pH 6.8 and 10 mM EDTA, with a 30-sec sonication. Equal protein concentrations (30 µg) were separated on 12% SDS-PAGE, and transferred onto a PVDF membranes. The membranes were blocked with with blocking buffer (PBS containing 3%BSA and 0.1% sodium azide), for the reason that the molecular weight of Cx isoforms (37, 40, 43) and β-actin (42KD) are very close, then incubated for 1 h at room temperature with the following primary antibodies: rabbit polyclonal anti-Cx37 antibody (rabbit polyclonal Cx37 antibody raised against human; Cat. No. SAB4501180, Sigma-Aldrich), anti-Cx40 antibody (rabbit polyclonal Cx40 antibody raised against human; Cat. No. SAB1304973, Sigma-Aldrich) and anti-Cx43 antibody (rabbit polyclonal Cx43 antibody raised against human; Cat. No. C6219, Sigma-Aldrich) (1:1000) and anti-β actin antibody (rabbit polyclonal antibody raised against human; Cat. No. SAB5500001, Sigma-Aldrich) (1:1000). The membranes were then incubated with horseradish peroxidase-conjugated goat anti-rabbit immunoglobulin G (Cat. No. 6154, Sigma-Aldrich) (Zhang et al. 2021), protein expression detected via enhanced chemiluminescence, and bands quantified using Bio-Rad Image Lab software. Expression of target proteins was normalized to that of *β*-actin.

### Statistical analysis

All of data were statistically analyzed using SPSS 13.0 software and presented as means ± standard deviations (SD) of the mean. Differences between groups (n = 6) were analyzed using the t-test, at significance levels of *P < 0.05 and **P < 0.01.

## Results

### Simvastatin prevents TNF-α-induced GJIC activity inhibition in HUVECs

**Figure 2.**
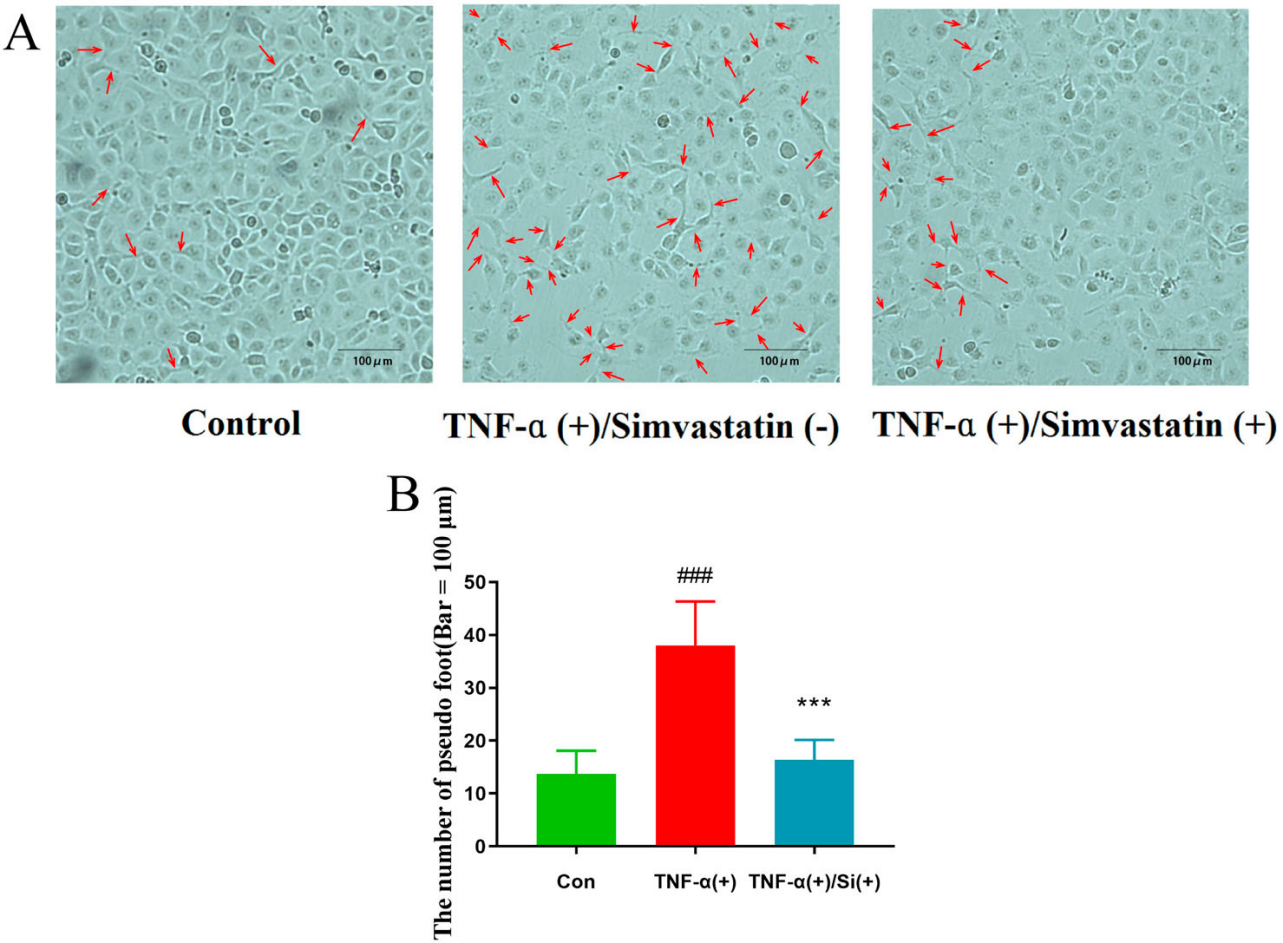
Profiles of cell morphology (200× magnification). Pseudopod increased in the TNF-α treatment, relative to the control group, while pseudo foots were significantly lower in the TNF-α combined with simvastatin relative to the TNF-α alone group (red arrows). Contrast phase microscope 100×. B: Analysis of the number of pseudo foot in each group. Error bars represent SD, *n* = 6, ^###^*P *< 0.001 versus the control group, ***P < 0.001 versus the TNF-α treatment group. Bar = 100 μm.

**Figure 3.**
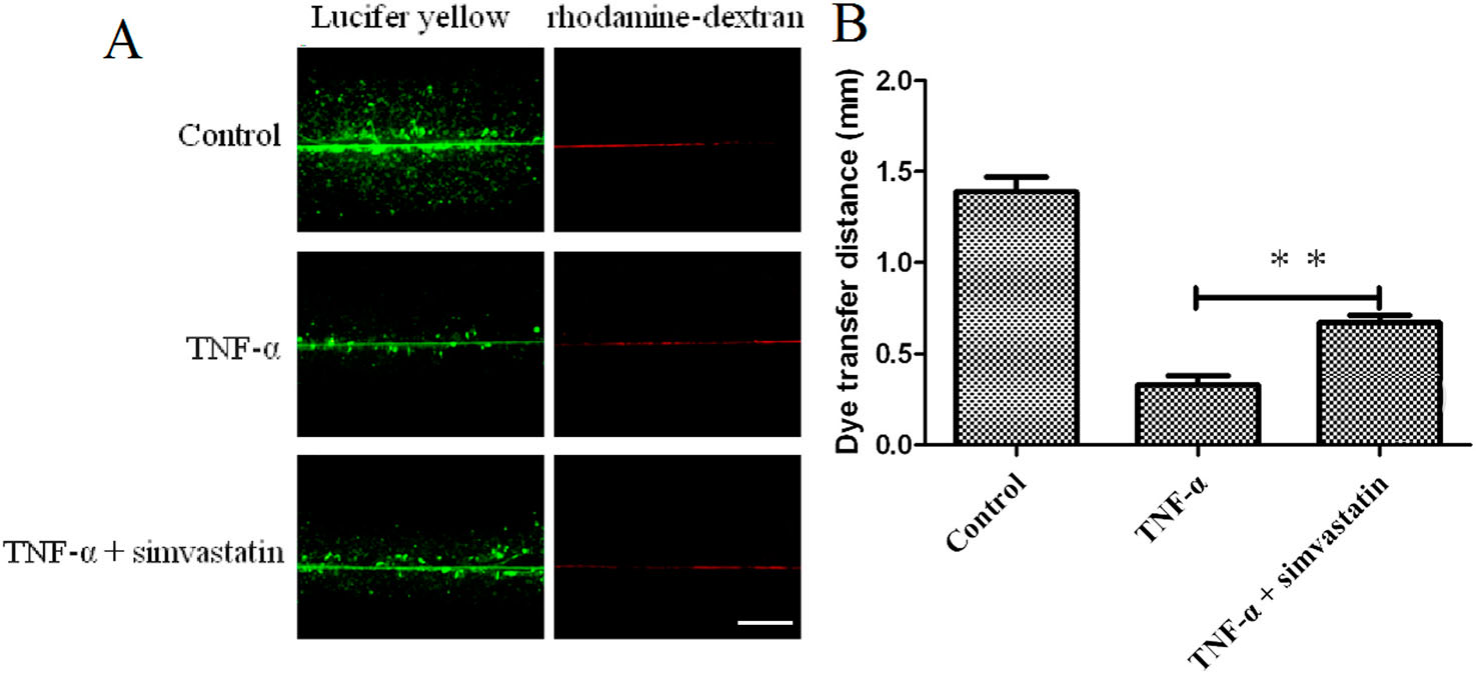
Simvastatin suppresses TNF-α-induced inhibition of GJIC activity in HUVECs, based on the Scrape loading assay. A: Fluorescence micrographs with Lucifer yellow (LY, MW: 457, 25 Da) and rhodamine-dextran (RD, MW: 10 kDa). (Original magnification ×10). B: The distance between the dye transfer front and the scrape line in each group. Error bars represent SD (*n* = 6), **P < 0.01 versus the TNF-α treatment group. Bar = 40 μm.

Observation of cells under white light (200× magnification) revealed a higher number of pseudopods was increased in the TNF-α treatment group compared to . On the other hand, pseudo foots were significantly lower in TNF-α combined with simvastatin treatment, relative to the group treated with TNF-α alone (Figure 2). Confluent cultures were scraped and incubated with the GJIC-permeable fluorescent Lucifer yellow (LY, green) and the GJIC-impermeable fluorescent rhodamine-dextran (RD, red) dyes (Figure 3). HUVEC colonies in the control group exhibited extensive LY diffusion, at an average LY transfer distance of 1.39 ± 0.08 mm. TNF-α treatment significantly reduced LY transfer among cells to an average of 0.33 ± 0.05 mm. However, simvastatin treatment inhibited this effect resulting in an average LY transfer distance of 0.67 ± 0.04 mm.

### Effect of simvastatin on GJIC activity and expression of Cx37, Cx40 and Cx43 proteins

**Figure 4.**
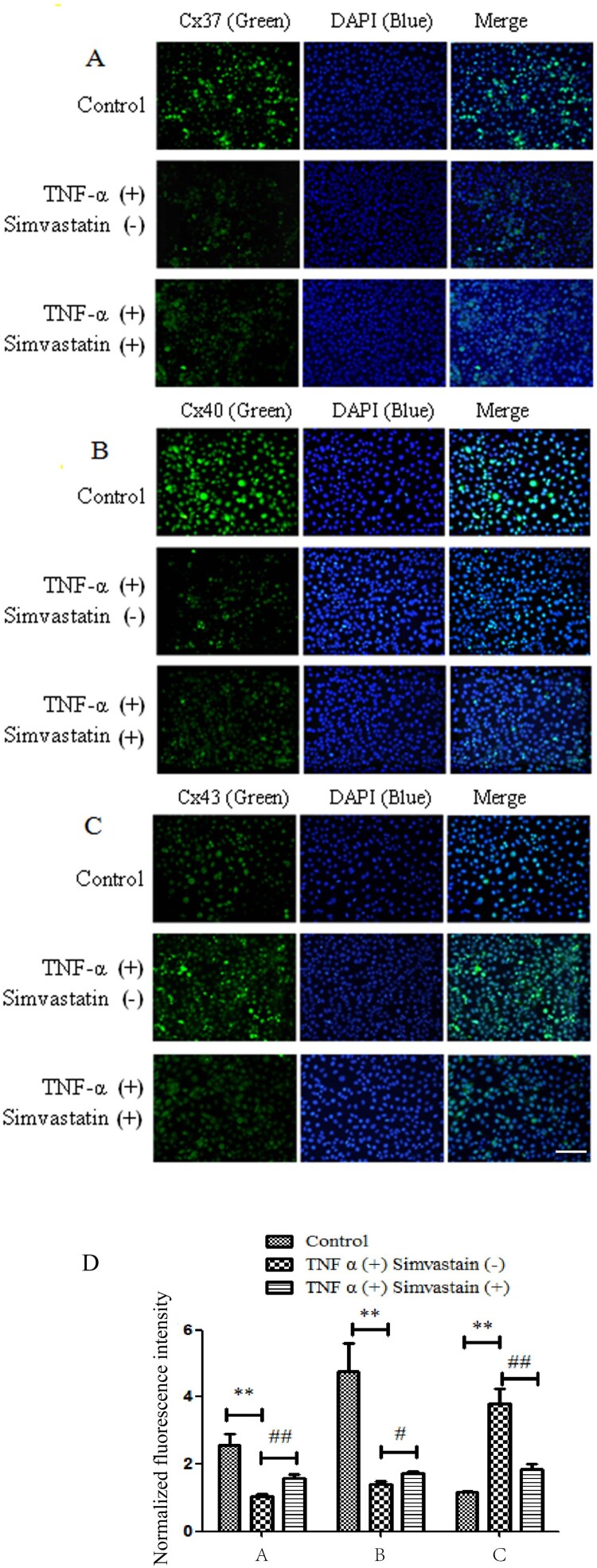
Immunofluorescence results showing effect of simvastatin on GJIC targeting Cx37, Cx40 and Cx43. Cx37 (A) and Cx40 (B) expression was downregulated in gap junctions of HUVECs treated with TNF-α relative to the control group. Treatment of HUVECs with simvastatin upregulated Cx37 and Cx40 expression. Similarly, HUVECs treated with TNF-α exhibited Cx43 up-regulation (C) although simvastatin reversed this phenomenon. D: Quantitative comparison of FITC staining intensity normalized to control. *n* = 6, *P < 0.05, **P < 0.01 *vs.* Control; #*P* < 0.05, ##*P* < 0.01 *vs.* TNF-α (+) Simvastain (−) group. Bar = 30 μm.

Results from SL/DT assay showed that simvastatin treatment suppressed TNF-α-induced inhibition of GJIC activity in HUVECs, and affected expression of Cx37, Cx40 and Cx43 in endothelial cells. These proteins play an important role in GJIC. Immunofluorescence results showed that TNF-α treatment downregulated expression of Cx37 and Cx40 gap junctions in HUVECs relative to those in the control group. However, exposure of HUVECs simvastatin upregulated Cx37 and Cx40 expression. In addition, TNF-α treatment upregulated while exposure to simvastatin downregulated Cx43 expression in HUVECs (Figure 4).

### Effect of simvastatin expression of Cx37, Cx40 and Cx43 mRNAs in HUVECs

**Figure 5.**
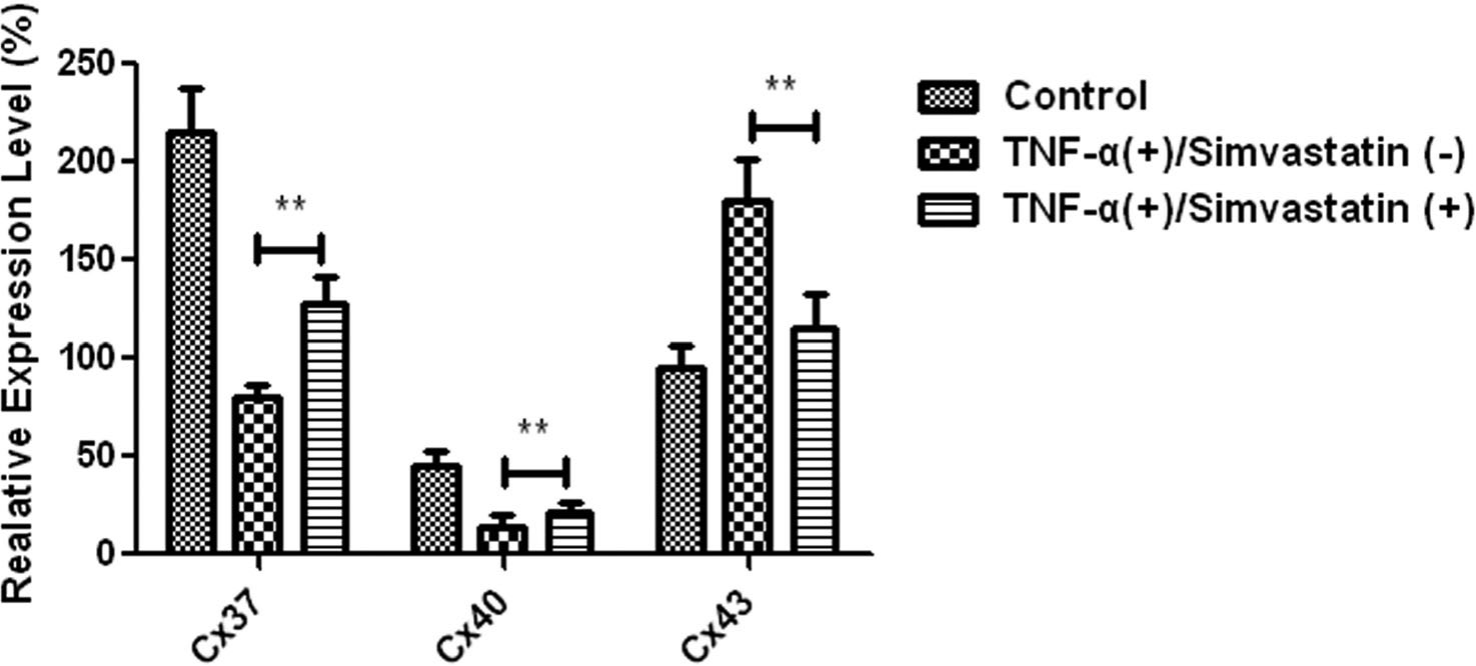
Differential expression of Cx37, Cx40 and Cx43 mRNAs in HUVECs across groups. Cx37 and Cx40 had a 2-fold downregulation, whereas Cx43 was upregulated relative to the control group. Cx37 and Cx40 were significantly upregulated in HUVECs treated with TNF-α in combination with simvastatin relative to those treated with TNF-α alone. Cx43 was significantly downregulated. Data presented are means ± SEM, *n* = 6, **P < 0.01 versus only TNF-α treatment group.

Real-time PCR results showed that Cx37, Cx40 and Cx43 were expressed in HUVECs in both control and TNF-α treatment groups, although expression levels Significantly differed. Particularly, Cx37 and Cx40 mRNAs showed a 2-fold downregulation, while Cx43 was upregulated in TNF-α treatment relative to the control group. However, treatment of HUVECs with TNF-α in combination with simvastatin (5 µM) significantly upregulated Cx37 (80 ± 7 vs 128 ± 14, *P *< 0.01) and Cx40 (14 ± 3 vs 21 ± 6, *P *< 0.01) relative to those treated with TNF-α alone. On the other hand, Cx43 was significantly downregulated in the combination relative to the TNF-α alone group (180 ± 21 vs 115 ± 15, *P *< 0.01) (Figure 5).

### Effect of simvastatin on expression of Cx37, Cx40 and Cx43 proteins in HUVECs

**Figure 6.**
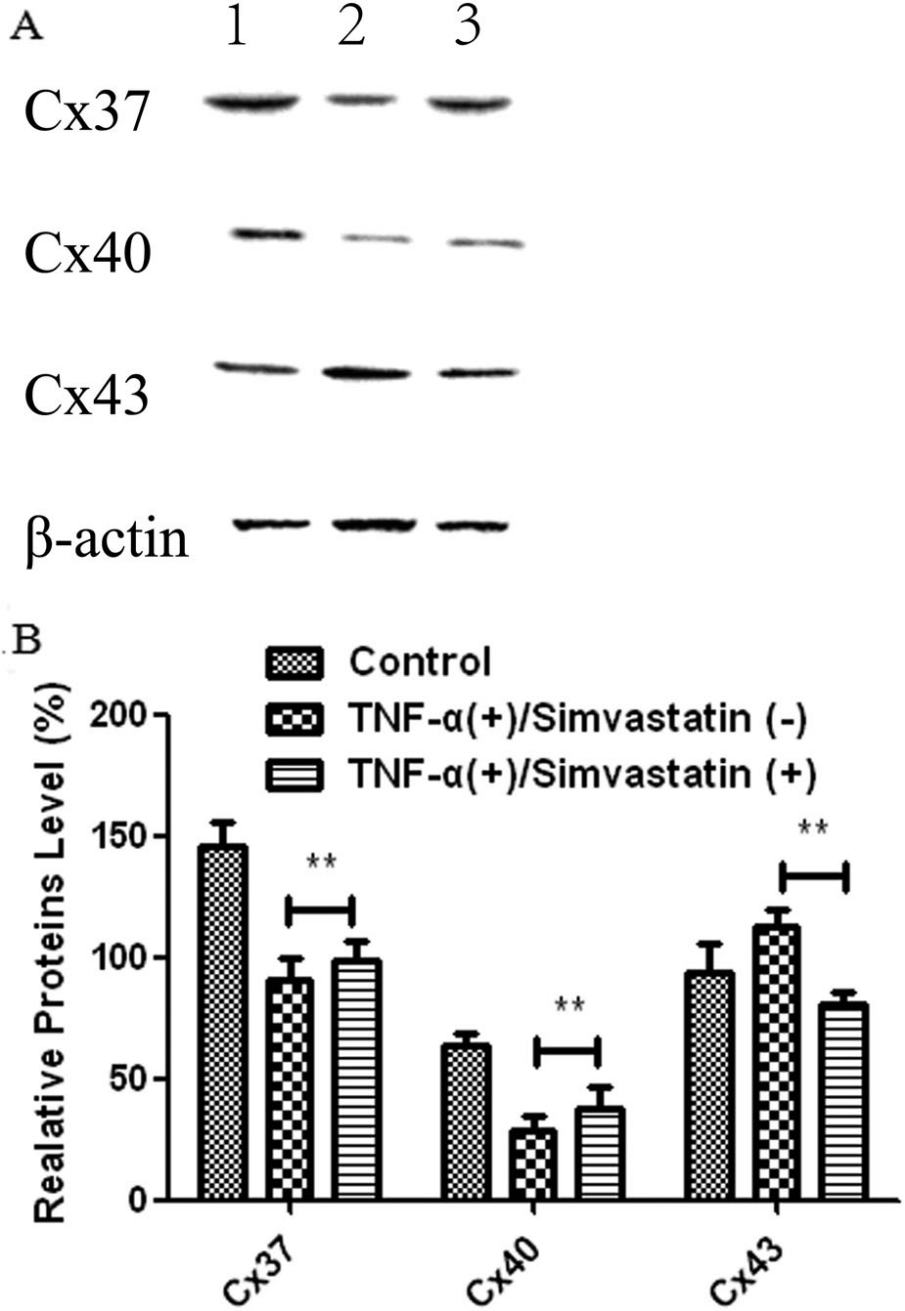
Cx37, Cx40 and Cx43 proteins were differentially expressed in HUVECs in different treatment groups. A: Western blots. 1: Control, 2: TNF-α (+)/Simvastatin (−), 3: TNF-α (+)/Simvastatin (+). B: qRT-PCR results showing relative transcript levels. Data presented are means ± SEM, *n*** **= 6, **P < 0.01 versus only TNF-α treatment group.

Western blots showed that treatment of HUVECs with TNF-α for 24 h significantly downregulated expression of Cx37 (145.85 ± 10.01 vs 90.94 ± 8.58) and Cx40 (64.25 ± 4.89 vs 28.50 ± 6.54) but upregulated that of Cx43 (93.70 ± 12.36 vs 112.69 ± 7.21) proteins relative to the control group. However, these patterns of expression were reversed after simvastatin treatment. In addition, Cx37 (90.94 ± 8.58 vs 98.75 ± 7.66) and Cx40 (28.50 ± 6.54 vs 37.69 ± 9.58) proteins were significantly upregulated, while Cx43 was significantly downregulated (112.69 ± 7.21 vs 81.03 ± 5.28) in HUVECs treated with TNF-α in combination with simvastatin, relative to those treated with TNF-α alone (Figure 6).

## Discussion

Gap junctions, first identified in the 1960s, have been shown to play an important role in maintenance of normal cellular functions (Qu et al. 2014; Evans WH. 2015), while gap junction intercellular contact (GJIC) has been associated with inflammatory-induced increase in vascular permeability (O’Donnell et al. 2014). Statins are the most commonly used lipid-modifying medications in the world (Adams et al. 2012). In the gap junction, statins have been shown to exert pharmacological activity against atherosclerosis, neointimal hyperplasia, arrhythmia, and cancer (Barros et al. 2011; Mihos et al. 2014; Qu et al. 2014). In fact, some researchers have hypothesized that simvastatin’s pleiotropic effects could be related to an increase in GJIC seen in the current research (Wang et al. 2013). However, statins’ effect in lowering endothelial GJIC and subsequent prevention of activities of inflammatory mediators remain unclear. Therefore, in the present study, we activated HUVECs by treating them with TNF-α alone, or in combination with simvastatin, then evaluated their effects on vascular endothelial cell GJIC.

Results of the SL/DT assay showed that TNF-α significantly suppressed GJIC activity, which was consistent with previous studies that have shown that TNF-α could inhibit GJIC activity in vascular endothelial cells, liver epithelial cells and human corneal fibroblasts (Kimura et al. 2013; Mihos et al. 2014; Okamoto et al. 2014). Additional evidences have associated alterations of gap junctions with cardiac abnormalities, such as arrhythmia, necrosis and apoptosis of cardiomyocytes in animals (Chen et al. 2010; Marsh et al. 2016). Results of the present study further showed that treating HUVECs with TNF-α in combination with simvastatin significantly increased their GJIC activity indicating simvastatin’s beneficial effects (Marsh et al. 2016).

Results from a previous study showed that simvastatin improved sensitivity of Leydig tumor cells to chemotherapeutic toxicity by enhancing gap junction functions (Wang et al. 2015). Notably, gap junctions are composed of transmembrane proteins, named connexins (Cxs) (Thevenin et al. 2013). Previous studies have identified various types of proteins in the gap junction, key among them being Cx37, Cx40 and Cx43, which in arterial walls regulate the progression of atherosclerosis (Pfenniger et al. 2013). In contrast to the atheroprotective roles of Cx37 and Cx40, Cx43 merely acts as an atherogenic protein (Morel et al. 2014). To further clarify the simvastatin’s regulatory role on gap junction functions, we performed immunofluorescence, real-time PCR and western blot assays to analyze expression of Cx37, Cx40 and Cx43 mRNAs and proteins. Results all showed that simvastatin treatment upregulates Cx37 and Cx40 mRNAs and proteins. Previous studies have shown that mouse aortic endothelial gap junctions and connexins are downregulated during long-term hyperlipidemia, whereas short-term treatment with simvastatin leads to recovery of Cx37 but not Cx40 expression (Yeh et al. 2003). Other research evidences have shown that in the presence of statins, formation of the neointima induced by balloon injury is reduced along with downregulation of Cx40 expression at the site of injury (Wang et al. 2005). Results of the present study showed that both Cx37 and Cx40 were upregulated, possibly due to different experimental methods and models. Our results were consistent with the findings of Hou et al. (2008) who showed that simvastatin significantly reversed the downregulation in endothelial Cx37 and Cx40 expression in diabetic mice. Downregulation of Cx43, a cardiovascular risk marker and a therapeutic target, was shown to inhibit atherosclerotic lesion formation in low-density lipoprotein receptor-deficient mice (Wei et al. 2013). To investigate the underlying mechanism of simvastatin-mediated suppression of GJIC activity dysfunction, we analyzed Cx43 expression and found that treatment of HUVECs with TNF-α in combination with simvastatin downregulated expression of both its mRNA and protein. This was consistent with previous studies that have shown that simvastatin is a functional factor with the ability to attenuate the additive effects of TNF-α and IL-18 in Cx43 up-regulation and over-proliferation of cultured aortic smooth muscle cells (Lin et al. 2013).

Overall, these marked changes indicate that simvastatin exerts its regulatory effects on gap junction functions by upregulating Cx37 and Cx40 but downregulating Cx43 expression. These findings may also fortify the rationale underlying the atheroprotective mechanism of simvastatin therapy. Cx43 is the most predominant gap junction protein among various types of connexins, and has been shown to play essential roles in coordinating activities of cardiovascular tissues, key among them being selective inhibition of Cx43 hemichannels by Gap19 and its impact on myocardial ischemia/reperfusion injury %J Basic Research in Cardiology (Wang et al. 2013). Generally, time and dose are also important factors affecting simvastatin effect. Moreover, effects of other types of cell models, such as vascular smooth muscle cell (VSMC) and macrophages, as well as the intercellular interaction between vascular endothelial cell (VEC) and VSMC, VEC and macrophage are expected to be evaluated in further studies. Future explorations, using animal models and human subjects, are expected to validate these findings.

To gain more mechanistic insights into the underlying biological mechanism, analyzing high-throughput molecular measurements at the functional level was selected. Especially, the knowledge base-driven pathway analysis is becoming the first choice for many investigators, which mainly exploit pathway knowledge in public repositories (Du et al., 2016). KEGG pathway enrichment results showed that the targeted genes which were regulated by connexins were involved in many pathways, including EGFR tyrosine kinase inhibitor resistance, Endocrine resistance, and those regulating MAPK, ErbB, Ras, Rap1, and Phospholipase D signaling, among others. These signaling pathways could be potential targets for future research.

Numerous studies have described simvastatin’s effect on cadherin. Notably, adhesion junctions are extensively distributed in microvessels, so few focus beneficial effect of Simvastatin on tight junction (Zapolska-Downar et al. 2004; Khaidakov et al. 2009). These findings suggest that statins modulate cell–cell adhesion, through VE-cadherin stimulation, thereby diminishing cellular proliferation and migration. Since tight junction interspersed with adhesion junction also play an important role in maintaining vascular endothelial cell integrity, we sought to demonstrate that simvastatin can also affect the tight junction. Furthermore, we hypothesize that cadherin interacts with the tight junction, although the specific target proteins and underlying mechanism of action remain unknown. These require further explorations.

## Data Availability

Raw data supporting the conclusions of this article will be made available by the authors, without undue reservation, to any qualified researcher.

## Abbreviations

**Abbreviations:** TNF-α Tumor necrosis factor-alpha, GJIC Gap junction intercellular communication, Cx37 Connexin 37, Cx40 Connexin 40, Cx43 Connexin 43
